# Constructing a universe for the setoid model

**DOI:** 10.1007/978-3-030-71995-1_1

**Published:** 2021-03-23

**Authors:** Thorsten Altenkirch, Simon Boulier, Ambrus Kaposi, Christian Sattler, Filippo Sestini

**Affiliations:** 8 University of Oxford, Oxford, UK; 9 Sorbonne Université - LIP6, Paris, France; 10 School of Computer Science, University of Nottingham, Nottingham, UK; 11 Inria, Nantes, France; 12 Eötvös Loránd University, Budapest, Hungary; 13 Chalmers University of Technology, Gothenburg, Sweden

**Keywords:** type theory, function extensionality, univalence, setoid model, induction-recursion, induction-induction

## Abstract

The setoid model is a model of intensional type theory that validates certain extensionality principles, like function extensionality and propositional extensionality, the latter being a limited form of univalence that equates logically equivalent propositions. The appeal of this model construction is that it can be constructed in a small, intensional, type theoretic metatheory, therefore giving a method to boostrap extensionality. The setoid model has been recently adapted into a formal system, namely Setoid Type Theory (SeTT). SeTT is an extension of intensional Martin-Löf type theory with constructs that give full access to the extensionality principles that hold in the setoid model.

Although already a rich theory as currently defined, SeTT currently lacks a way to internalize the notion of type beyond propositions, hence we want to extend SeTT with a universe of setoids. To this aim, we present the construction of a (non-univalent) universe of setoids within the setoid model, first as an inductive-recursive definition, which is then translated to an inductive-inductive definition and finally to an inductive family. These translations from more powerful definition schemas to simpler ones ensure that our construction can still be defined in a relatively small metatheory which includes a proof-irrelevant identity type with a strong transport rule.

* Supported by USAF grant FA9550-16-1-0029.
† Supported by ERC Starting Grant CoqHoTT 637339.
‡ Supported by the Bolyai Fellowship of the Hungarian Academy of Sciences (BO/00659/19/3) and by the "Application Domain Specific Highly Reliable IT Solutions" project that has been implemented with support from the National Research, Development and Innovation Fund of Hungary, financed under the Thematic Excellence Programme TKP2020-NKA-06 funding scheme.
§ Supported by USAF grant FA9550-16-1-0029 and Swedish Research Council grant 2019-03765.

## 1 Introduction

Intuitionistic type theory is a formal system designed by Per Martin-Löf to be a full-fledged foundation in which to develop constructive mathematics [23,24]. A central aspect of type theory is the coexistence of two notions of equality. On the one hand definitional equality, the computational equality that is built into the formalism. On the other hand "propositional" equality, the internal notion of equality that is actually used to state and prove equational theorems within the system. The precise balance between these two notions is at the center of type theory research; however, it is generally understood that to properly support formalization of mathematics, one should aim for a notion of propositional equality that is as *extensional* as possible.

Two extensionality principles seem particularly desirable, since they arguably constitute the bare minimum for type theory to be comparable to set theory as a foundational system for set-level mathematics, in terms of power and ergonomics. One is function extensionality (or *funext*), according to which functions are equal if point-wise equal. Another is propositional extensionality (or *propext*), that equates all propositions that are logically equivalent.

Type theory with equality reflection, also known as *extensional type theory* (ETT) does support extensional reasoning to some degree, but unfortunately equality reflection makes the problem of type-checking ETT terms computationally unfeasible: it is undecidable.

On the other hand, *intensional type theory* (ITT) has nice computational properties like decidable type checking that can make it more suitable for computer implementation, but as usually defined (for example, in [23]) it severely lacks extensionality. It is known from model constructions that extensional principles like funext are consistent with ITT. Moreover, ITT extended with the principle of *uniqueness of identity proofs* (UIP) and funext is known to be as powerful as ETT [19]. We could recover the expressive power of ETT by adding these principles to ITT as axioms, however destroying some computational properties like canonicity.

What we would like instead is a formulation of ITT that supports extensionality, while retaining its convenient computational behaviour. Unfortunately, canonicity for Martin-Löf's inductively defined identity type says that if two terms are propositionally equal in the empty context, then they are also definitionally equal. This rules out function extensionality. The first step towards a solution is to give up the idea of propositional equality as a single inductive definition given generically for arbitrary types. Instead, equality should be *specific* to each type former in the type theory, or in other words, every type former should be introduced alongside an explanation of what counts as equality for its elements.

This idea of pairing types together with their own equality relation goes back to the notion of *setoid* or *Bishop set*. Setoids provide a quite natural and useful semantic domain in which to interpret type theory. The first setoid model was constructed to justify function extensionality without relying on funext in the metatheory [18]. Moreover, it was shown by Altenkirch [4] that if the model construction is carried out in a type theoretic metatheory with a universe of strict (definitionally proof-irrelevant) propositions, it is possible to define a univalent universe of propositions satisfying propositional extensionality. The setoid model thus satisfies all the extensionality principles that we would like to have in a set-level type theory [5] . The question is whether there exists a version of intensional type theory that supports setoid reasoning, and hence the forms of extensionality enabled by it.

This question was revisited and answered in Altenkirch et al. [5]. In this paper, the authors define Setoid Type Theory (SeTT), an extension of intensional Martin-Löf type theory with constructs for setoid reasoning, where funext and propext hold by definition. SeTT is based on the *strict* setoid model of Altenkirch[6], which makes it possible to show consistency via a syntactic translation. This is in contrast with other type theories based on the setoid model, like Observational Type Theory [9] and XTT [28], which instead rely on ETT for their justification. A major property of SeTT is thus to illustrate how to bootstrap extensionality, by translation into a small intensional core.

SeTT as defined in [5] is already a rich theory, but its introspection capabilities are currently lacking, as its universes are limited to propositions. We would like to internalise the notion of type in SeTT, thus extending the theory with a universe of setoids. This goal brings up several questions, one of which has to do with the notion of equality with which the universe should come equipped: the universe of setoids is itself a setoid (as any type is) so it certainly cannot be univalent, since setoids lack the necessary structure. Another issue is the way such universe can be justified by the setoid model, and in particular what principles are needed in the metatheory to do so.

*Contributions* This paper documents our work towards the construction of a universe of setoids inside the setoid model, and tries to answer these and other questions related to the design and implementation of this construction. Our main contribution is the construction of the universe in the model; this is given in steps, first as an inductive-recursive definition, which is then translated to an inductive-inductive definition, and subsequently to an inductive type. As a consequence, we show that we only need to assume indexed W-types and proof-irrelevant identity types in the metatheory (along with some obligatory basic tools like $\Sigma$ and $\Pi$ types) to construct the universe.

The universe constructions presented in this paper are, to our knowledge, the first examples of two kinds of data type reductions in an intensional metatheory: the first involving an inductive-recursive type which includes strict propositions, and the second involving an infinitary inductive-inductive type.

Finally, the mathematical contents of this paper have been formalized in the proof-assistant Agda (see [10]).

*Structure of the paper* We begin by describing the metatheory that we will use throughout the paper, in Section 2. In Section 3, after briefly recalling *cate-*

[5] In the sense of HoTT we mean a type theory limited to h-sets.

[6] A *strict* model is one where every equation holds definitionally.

*gories with families* as an abstract notion of models of type theory, we outline Altenkirch's setoid model as given in [5]. We then briefly discuss the rules of Setoid Type Theory in Section 3.2.

In Section 4 we discuss the setoid model and various design choices related to it. We then recall inductive-recursive universes, and the way they can be equivalently defined as a plain inductive definition, in Section 4.1. We then provide, in Section 4.2, a first complete definition of the setoid universe using a special form of induction-recursion. This form of induction-recursion is not known to be reducible to plain inductive types. Then we describe an alternative definition of the universe in Section 4.3, that does not rely on induction-recursion but instead on infinitary induction-induction. This inductive-inductive encoding of the universe is obtained from the inductive-recursive one, inspired by the method of Section 4.1. We end the series of universe constructions with Section 4.4, where we outline a purely inductive definition of the setoid universe, obtained from the inductive-inductive one.

#### 1.1 Related work

The setoid model was first described in [18] in order to add extensionality principles to Type Theory such as function extensionality and propositional extensionality. A strict variant of the setoid model was given in [4] using a definitionally proof-irrelevant universe of propositions. Recently, support for such a universe was added to the proof-assistants Agda and Coq [17], allowing a full formalization of Altenkirch's setoid model. Setoid Type Theory (SeTT) is a recently developed formal system derived from this model construction [5]. Observational Type Theory (OTT) [9] is a syntax for the setoid model differing from SeTT in the use of a different notion of heterogeneous equality. Moreover, the consistency proof for OTT relies on Extensional Type Theory, whereas for SeTT it is obtained via a syntactic translation. XTT [28] is a cubical variant of OTT where the equality type is defined using an interval pretype [7] . XTT's universes support universe induction, whereas it is left open whether the construction presented here supports this principle. Palmgren and Wilander [27] construct a setoid universe using a translation into constructive set theory. Palmgren [26] constructs an encoding of ETT in ITT through Aczel's encoding of set theory in type theory [3]. He uses type theory as a language for his formalisation but his construction is set-theoretic in nature. Setoids are utilized to encode sets as arbitrarily branching well-founded trees quotiented by bisimulation. His notion of family of setoids does not use strict propositions and it has a weaker form of proof irrelevance which seems to be not enough to obtain a model of SeTT.

The principle of propositional extensionality in the setoid model is an instance of Voevodsky's univalence axiom [29]. The cubical set model is a constructive model justifying this axiom [11]. A type theory extracted from this model is Cubical Type Theory [13]. The relationship between the cubical set

[7] To quote one of the referees: *the fact that the interval is a pretype is but the easiest part of the story.*

model and cubical type theory is similar to that between the setoid model and SeTT. Compared to cubical type theories, SeTT has the advantage that the equality type satisfies more definitional equalities. For instance, whereas in cubical type theory equality of functions is isomorphic to pointwise equality, in SeTT the isomorphism is replaced by a definitional equality. SeTT is also a syntactically straightforward extension of Martin-Löf Type Theory, that does not require exotic objects like the interval pretype. In turn, the obvious advantage of cubical type theory is that it is not limited to setoids.

An exceptional aspect of the metatheory used in this paper is the presence of a proof-irrelevant identity type with a strong transport rule allowing to eliminate into arbitrary types. In [1], Abel gives a proof of normalization for the Logical Framework extended with a similar proof-irrelevant equality type. Abel and Coquand show in [2] that the combination of impredicativity with a strong transport rule results in terms that fail to normalize but this is irrelevant in our setting.

### 2 $\mathsf{MLTT}^{\mathbf{Prop}}$

This section describes $\mathsf{MLTT}^{\mathbf{Prop}}$, our ambient metatheory. We employ Agda notation to write down $\mathsf{MLTT}^{\mathbf{Prop}}$ terms throughout the paper.

One of the main appeals of Altenkirch's setoid model is that it can justify several useful extensionality principles while being defined in a small intensional metatheory. We tried to stay true to this idea when figuring out the necessary metatheoretical tools for the universe construction in this paper. In particular, we wanted to avoid having to assume strong definition schemas that go beyond inductive families. $\mathsf{MLTT}^{\mathbf{Prop}}$ is thus an intensional type theory in the style of Martin-Löf type theory.

We have sorts $\mathbf{Type}_i$ of types and $\mathbf{Prop}_i$ of strict propositions for $i \in \{0, 1\}$. Here, $i = 0$ means "small" (and we will omit the subscript) and $i = 1$ means "large". We have implicit lifting from $i = 0$ to $i = 1$, but do not assume type formers are preserved. $\mathbf{Type}_1$ has universes for $\mathbf{Type}$ and $\mathbf{Prop}$. We do not distinguish notationally between universes and sorts. We continue to describe only the case $i = 0$; everything introduced has an analogue at level $i = 1$. Propositions lift to types via $\mathsf{Lift} : \mathbf{Prop} \to \mathbf{Type}$, with constructor $\mathsf{lift} : \{P : \mathbf{Prop}\} \to P \to \mathsf{Lift}\ P$ and destructor $\mathsf{unlift} : \{P : \mathbf{Prop}\} \to \mathsf{Lift}\ P \to P$.

We have standard type formers $\Pi, \Sigma, \mathsf{Bool}, \mathbf{0}, \mathbf{1}$ in $\mathbf{Type}$. $\Sigma$-types are defined negatively by pairing $-,-$ and projections $\pi_1, \pi_2$. We have definitional $\eta$-rules for $\Pi$-, $\Sigma$-, $\mathbf{1}$-types. We also require indexed W-types, both in $\mathbf{Type}$ and $\mathbf{Prop}$: $W_\Box : (S : I \to \mathbf{Type}) \to ((i : I) \to S\ i \to I \to \mathbf{Type}) \to I \to \Box$ where $\Box \in \{\mathbf{Type}, \mathbf{Prop}\}$. The elimination principle of $W_{\mathbf{Prop}}$ only allows defining functions into elements of $\mathbf{Prop}$. From $W_{\mathbf{Prop}}$ we can define propositional truncation $\|-\| : \mathbf{Type} \to \mathbf{Prop}$, with constructor $|-| : \{A : \mathbf{Type}\} \to A \to \|A\|$ and eliminator $\mathsf{elim}_{\|-\|} : \{P : \mathbf{Prop}\} \to (A \to P) \to \|A\| \to P$.

In addition to type formers in $\mathbf{Type}$, we will need the propositional versions of $\mathbf{0}$, $\mathbf{1}$, $\Pi$, and $\Sigma$. The latter three can be defined from their $\mathbf{Type}$ counterparts via truncation. That is, given $P : \mathbf{Prop}$ and $Q : P \to \mathbf{Prop}$:

$$\begin{aligned} \mathbf{1}_{\mathbf{Prop}} &:\equiv \|\mathbf{1}\| \\ \Pi_{\mathbf{Prop}}\ P\ Q &:\equiv \|\Pi\ (\mathsf{Lift}\ P)\ (\mathsf{Lift} \circ Q \circ \mathsf{unlift})\| \\ \Sigma_{\mathbf{Prop}}\ P\ Q &:\equiv \|\Sigma\ (\mathsf{Lift}\ P)\ (\mathsf{Lift} \circ Q \circ \mathsf{unlift})\| \end{aligned}$$

We assume that we have $\mathbf{0}_{\mathbf{Prop}} : \mathbf{Prop}$ together with $\mathsf{exfalso}_{\mathbf{Prop}} : \{A : \mathbf{Type}\} \to \mathbf{0}_{\mathbf{Prop}} \to A$.

Finally, we will assume an identity type in the style of Martin-Löf's inductive identity type. The main difference is that our identity type is a **Prop**-valued relation. We have a transport combinator $\mathsf{transp}$ from which $\mathsf{J}$ is derivable.

$$\begin{aligned} \mathsf{Id} &: \{A : \mathbf{Type}\} \to A \to A \to \mathbf{Prop} \\ \mathsf{refl} &: \{A : \mathbf{Type}\}(a : A) \to \mathsf{Id}\ a\ a \\ \mathsf{transp} &: \{A : \mathbf{Type}\}(C : A \to \mathbf{Type})\{a_0\ a_1 : A\} \to \mathsf{Id}\ a_0\ a_1 \to C\ a_0 \to C\ a_1 \end{aligned}$$

with $\mathsf{transp}\ C\ \{x\}\ \{x\}\ e\ u \equiv u$. The $\mathsf{transp}$ combinator provides a strong elimination principle allowing to eliminate a strict proposition (the identity type) into arbitrary types. We only use this identity type in Section 4.4. For the rest of our constructions, the traditional Martin-Löf's identity type suffices.

#### 2.1 Formalization

A universe of strict propositions has been recently added to the Agda proof assistant [17], making *most* of $\mathsf{MLTT}^{\mathbf{Prop}}$ a subset of Agda, with the exception of the proof-irrelevant identity type. Most of the universe constructions presented here have been formalized and proof-checked using Agda, with the proof-irrelevant identity type and the strong transport rule added via postulates and rewriting. The formalization can be found in [10].

For convenience, we slightly deviate from $\mathsf{MLTT}^{\mathbf{Prop}}$ both in the paper and in the formalization, for instance by relying on pattern matching instead of eliminators, and using primitive versions of **Prop**-valued $\Pi$ and $\Sigma$ types instead of deriving them from truncation. We operate under the assumption that everything can be equivalently carried out in $\mathsf{MLTT}^{\mathbf{Prop}}$, although we have not fully checked all the necessary details.

### 3 Setoid model

By *setoid model* we mean a class of models of type theory where contexts/closed types are interpreted as setoids, i.e. sets with an equivalence relation, and dependent types are interpreted as dependent/indexed setoids. A setoid model was first given for intensional type theory by M. Hofmann [18], in order to provide a semantics for extensionality principles such as function and propositional extensionality.

Here we consider a similar model construction due to Altenkirch [4]. The peculiarity of this model is that it is presented in a type theoretic and intensional metatheory which includes a strict universe of propositions.

The setoid model thus defined validates function extensionality, a universe of propositions with propositional extensionality, and quotient types. Therefore, it provides a way to bootstrap and "explain" extensionality, since the model construction effectively gives an implementation of various extensionality principles in terms of a small, completely intensional theory.

#### 3.1 Setoid model as a CwF

The setoid model can be framed categorically as a category with families (CwF, [14]) with extra structure for the various type and term formers. The core structure of a CwF can be given as the following signature:

$$\begin{aligned}
\mathrm{Con} &: \mathbf{Type} \\
\mathrm{Ty} &: (\Gamma : \mathrm{Con}) \to \mathbf{Type} \\
\mathrm{Sub} &: (\Gamma\ \Delta : \mathrm{Con}) \to \mathbf{Type} \\
\mathrm{Tm} &: (\Gamma : \mathrm{Con}) \to \mathrm{Ty}\ \Gamma \to \mathbf{Type}
\end{aligned}$$

In our presentation of the setoid model, contexts are given by setoids, that is, types together with an equivalence relation. A key point of the model is that the equivalence relation is valued in **Prop** and is thus definitionally proof irrelevant.

$$\frac{\Gamma : \mathrm{Con}}{\begin{aligned}
|\Gamma| &: \mathbf{Type} \\
\Gamma^{\sim} &: |\Gamma| \to |\Gamma| \to \mathbf{Prop} \\
\mathsf{refl}\ \Gamma &: (\gamma : |\Gamma|) \to \Gamma^{\sim}\ \gamma\ \gamma \\
\mathsf{sym}\ \Gamma &: \forall\{\gamma_0\ \gamma_1\} \to \Gamma^{\sim}\ \gamma_0\ \gamma_1 \to \Gamma^{\sim}\ \gamma_1\ \gamma_0 \\
\mathsf{trans}\ \Gamma &: \forall\{\gamma_0\ \gamma_1\ \gamma_2\} \to \Gamma^{\sim}\ \gamma_0\ \gamma_1 \to \Gamma^{\sim}\ \gamma_1\ \gamma_2 \to \Gamma^{\sim}\ \gamma_0\ \gamma_2
\end{aligned}}$$

Types in a context $\Gamma$ are given by displayed setoids over $\Gamma$ with a fibration condition given by $\mathsf{coe}$, $\mathsf{coh}$. In the following, we sometimes omit implicit quantifications such as the $\forall\{\gamma_0\ \gamma_1\}$ in the type of $\mathsf{sym}\,\Gamma$.

$$\frac{A : \mathrm{Ty}\ \Gamma}{\begin{aligned}
|A| &: |\Gamma| \to \mathbf{Type} \\
A^{\sim} &: \{\gamma_0\ \gamma_1 : |\Gamma|\} \to \Gamma^{\sim}\ \gamma_0\ \gamma_1 \to |A|\gamma_0 \to |A|\gamma_1 \to \mathbf{Prop} \\
\mathsf{refl*} &: \{\gamma : |\Gamma|\}(a : |A|\gamma) \to A^{\sim}\ (\mathsf{refl}\ \Gamma\ \gamma)\ a\ a \\
\mathsf{sym*} &: \forall\{\gamma_0\,\gamma_1\,a_0\,a_1\}\{p : \Gamma^{\sim}\ \gamma_0\ \gamma_1\} \to A^{\sim}\ p\ a_0\ a_1 \to A^{\sim}\ (\mathsf{sym}\ \Gamma\ p)\ a_1\ a_0 \\
\mathsf{trans*} &: A^{\sim}\ p_0\ a_0\ a_1 \to A^{\sim}\ p_1\ a_1\ a_2 \to A^{\sim}\ (\mathsf{trans}\,\Gamma\,p_0\,p_1)\ a_0\ a_2 \\
\mathsf{coe} &: \Gamma^{\sim}\ \gamma_0\ \gamma_1 \to |A|\gamma_0 \to |A|\gamma_1 \\
\mathsf{coh} &: (p : \Gamma^{\sim}\ \gamma_0\ \gamma_1)(a : |A|\gamma_0) \to A^{\sim}\,p\,a\,(\mathsf{coe}\,A\,p\,a)
\end{aligned}}$$

This definition of types in the setoid model is different from the one in [4], but it is equivalent to it [12, Section 1.6.1]. The main difference here is in the use of a heterogeneous equivalence relation $A^{\sim}$ in the definition of types.

Substitutions are interpreted as functors between the corresponding setoids, whereas terms of type $A$ in context $\Gamma$ are sections of the type seen as a setoid fibration $\Gamma.A \to \Gamma$. Note that we only need to include components for the functorial action on objects and morphisms, since the functor laws follow from proof-irrelevance in the metatheory, and thus hold definitionally.

$$\frac{\sigma : \mathrm{Sub}\;\Gamma\;\Delta}{\begin{array}{l}|\sigma| : |\Gamma| \to |\Delta| \\ \sigma^{\sim} : \Gamma^{\sim}\;\rho_0\;\rho_1 \to \Delta^{\sim}\;(|\sigma|\rho_0)\;(|\sigma|\rho_1)\end{array}} \qquad \frac{t : \mathrm{Tm}\;\Gamma\;A}{\begin{array}{l}|t| : (\gamma : |\Gamma|) \to |A|\,\gamma \\ t^{\sim} : (p : \Gamma^{\sim}\;\gamma_0\;\gamma_1) \to A^{\sim}\;p\;(|t|\gamma_0)\;(|t|\gamma_1)\end{array}}$$

We can show that the setoid model validates the usual basic type formers ($\Pi, \Sigma$, etc.), function extensionality and a universe of strict propositions with propositional extensionality [4]. Note that we do not need identity types or inductive types (W-types) for this.

#### 3.2 Setoid Type Theory

The setoid model presented in the previous section is *strict*, that is, every equation of a CwF holds by definition in the semantics. One advantage of strict models is that they can be turned into *syntactic translations*, in which syntactic objects of the source theory are interpreted as their counterparts in another *target* theory. In the case of the setoid model, this gives rise to a *setoid translation*, where source contexts are interpreted as target contexts together with a target type representing the equivalence relation, and so on.[8]

A setoid translation is used in [5] to justify Setoid Type Theory (SeTT), an extension of Martin-Löf type theory (+ **Prop**) with equality types for contexts and dependent types that reflect the setoid equality of the model.

We recall the rules of SeTT that extend regular MLTT below, but with a variation: whereas the equality types in [5] are stated as elements of SeTT's internal universe of propositions, here we state the context equalities as elements of the external, metatheoretic universe **Prop**. This generalises the notion of model of SeTT thus making it easier to construct models. Equality on types is defined as before in [5].

We have a universe of propositions $\mathsf{Prop}$ defined as follows:

$$\frac{\Gamma : \mathrm{Con}}{\mathsf{Prop} : \mathrm{Ty}\;\Gamma} \qquad \frac{P : \mathrm{Tm}\;\Gamma\;\mathsf{Prop}}{\underline{P} : \mathrm{Ty}\;\Gamma} \qquad \frac{u : \mathrm{Tm}\;\Gamma\;\underline{P} \qquad v : \mathrm{Tm}\;\Gamma\;\underline{P}}{u \equiv v}$$

Equality type constructors for contexts and dependent types internalize the idea that every context and type comes equipped with a setoid equivalence relation. Note that **Prop** is the universe of the metatheory while $\mathsf{Prop}$ is the internal

[8] Semantically, this translation corresponds to a model construction, in particular a functor from the category of models of the target theory to the category of models of what will be Setoid Type Theory. Since the setoid translation is structural in the context component, we can work with models in the style of categories with families rather than contextual categories.

one. As in the model, equality for dependent types is indexed over context equality.

$$\dfrac{\Gamma : \mathrm{Con} \qquad \rho_0, \rho_1 : \mathrm{Sub}\ \Delta\ \Gamma}{\Gamma^{\sim}\ \rho_0\ \rho_1 : \mathbf{Prop}} \qquad \dfrac{\begin{array}{c} A : \mathrm{Ty}\ \Gamma \qquad \rho_{01} : \Gamma^{\sim}\ \rho_0\ \rho_1 \\ a_0 : \mathrm{Tm}\ \Delta\ A[\rho_0] \qquad a_1 : \mathrm{Tm}\ \Delta\ A[\rho_1] \end{array}}{A^{\sim}\ \rho_{01}\ a_0\ a_1 : \mathrm{Tm}\ \Delta\ \mathsf{Prop}}$$

We have rules witnessing that these are indeed equivalence relations. We only recall reflexivity:

$$\dfrac{\rho : \mathrm{Sub}\ \Delta\ \Gamma}{\mathsf{R}\ \rho : \Gamma^{\sim}\ \rho\ \rho} \qquad \dfrac{A : \mathrm{Ty}\ \Gamma \qquad \rho : \mathrm{Sub}\ \Delta\ \Gamma \qquad a : \mathrm{Tm}\ \Delta\ A[\rho]}{\mathsf{R}\ a : \mathrm{Tm}\ \Gamma\ \underline{A^{\sim}\ (\mathsf{R}\ \rho)\ a\ a}}$$

In addition, we also have rules representing the fact that every construction in SeTT respects setoid equality, so that we can transport along any such equality:

$$\dfrac{A : \mathrm{Ty}\ \Gamma \qquad \rho_0, \rho_1 : \mathrm{Sub}\ \Delta\ \Gamma \qquad p : \Gamma^{\sim}\ \rho_0\ \rho_1 \qquad a : \mathrm{Tm}\ \Delta\ A[\rho_0]}{\begin{array}{c} \mathsf{coe}_A\ p\ a : \mathrm{Tm}\ \Delta\ A[\rho_1] \\ \mathsf{coh}_A\ p\ a : \mathrm{Tm}\ \Delta\ \underline{A^{\sim}\ p\ a\ (\mathsf{coe}_A\ p\ a)} \end{array}}$$

Notably, equality types in SeTT compute definitionally on concrete type formers. In particular, they compute to their obvious intended meaning, so that an equality of pairs is a pair of equalities, an equality of functions is a map of equalities, and so on. From this, we get definitional versions of function and propositional extensionality.

We can easily recover the usual Martin-Löf identity type from setoid equality, with transport implemented via coercion.

$$\dfrac{A : \mathrm{Ty}\ \Gamma \qquad a_0, a_1 : \mathrm{Tm}\ \Gamma\ A}{\mathsf{Id}_A\ a_0\ a_1 :\equiv A^{\sim}\ (\mathsf{R}\ \Gamma)\ a_0\ a_1 : \mathrm{Tm}\ \Gamma\ \mathsf{Prop}}$$

$$\dfrac{P : \mathrm{Ty}\ (\Gamma.A) \qquad p : \mathrm{Tm}\ \Gamma\ (\mathsf{Id}\ A\ a_0\ a_1) \qquad t : \mathrm{Tm}\ \Gamma\ P[a_0]}{\mathsf{transp}\ P\ p\ t :\equiv \mathsf{coe}\ P\ (\mathsf{R}\ \mathsf{id}, p)\ t : \mathrm{Tm}\ \Gamma\ P[a_1]}$$

We can also derive Martin-Löf's J eliminator for this homogeneous identity type. The only caveat is that $\mathsf{transp}$ and the J eliminator do not compute definitionally on reflexivity.

### 4 Universe of setoids

As pointed out in the introduction, SeTT is seriously limited by the lack of a universes internalizing the notion of setoid. Our goal is to extend SeTT with a universe of setoids; since SeTT is a direct syntactic reflection of the setoid model, this essentially amounts to showing that a universe of setoids with the necessary structure and equations can be constructed within the setoid model. This opens several questions and possible design choices.

A first fundamental consideration has to do with the very definition of the setoid universe: as any type in the setoid model, this universe must be a setoid and thus come equipped with an equivalence relation. However, unlike the universe of propositions, a universe of setoids cannot be univalent, since this would force it to be a groupoid. The obvious choice is therefore to have a non-univalent universe, and instead define the universe's relation so that it reflects a simple syntactic equality of codes rather than setoid equivalence.

Another question has to do with the metatheoretic tools required to carry out the construction of the universe. In fact, one of the main aspects of the setoid model construction recalled in Section 3 and shown originally in [4] is that it can be carried out in a very small type theoretic metatheory, thus providing a way to reduce extensionality to a small intensional core. We would like to stay faithful to this ideal when constructing this setoid universe.

A known and established method for defining universes in type theory relies on induction-recursion (IR), a definition schema developed by Dybjer [15,16]. Inductive-recursive definitions can be found throughout the literature, from the already mentioned type theoretic universes, including the original formulation à la Tarski by Martin-Löf [24], to metamathematical tools like computability predicates.

Although universe constructions in type theory—including our own setoid universe—are naturally presented as inductive-recursive definitions, they may not necessarily require a metatheory with induction-recursion. In fact, it is possible to reduce some instances of induction-recursion to plain induction (more specifically, inductive families), including some universe definitions. We recall this reduction in Section 4.1.

Other design choices on the setoid universe are less essential, but still require careful consideration. For instance, one question is whether the setoid universe should support universe induction, thus exposing the inductive structure of the codes. Such an elimination principle is known to be inconsistent with univalence, although this is not an issue in our case; nevertheless it is not immediately clear if the elimination principle can be justified by the semantics, that is, if our encoding of the setoid universe in the model allows to define such a universe eliminator. The question arises because our final encoding of the setoid universe only supports a weak form of elimination, for reasons that are explained in Section 4.4. Although not currently needed, a stronger eliminator might be necessary to justify universe induction. This problem should not arise in the other encodings of the setoid universe (as given in Section 4.2 and Section 4.3).

Another design choice has to do with how the setoid universe relates to the other universes. One could provide a code for $\mathsf{Prop}$ in the setoid universe. Moreover, the setoid universes could form a hierarchy, possibly cumulative.

Yet another choice is whether to have two separate sorts, one for propositions and one for sets (with propositions convertible to sets) or a single sort of types (sets), with propositions given by elements of a universe of propositions, which is a (large) type. We have chosen to present the second option to fit with the standard notion of (unisorted) CwF. However, this has downsides: to even talk about propositions, we need to have a notion of large types. The first option is more symmetric: we can have parallel hierarchies for propositions and sets.

#### 4.1 Inductive-recursive universes

An inductive-recursive universe is given by a type of codes $\mathsf{U} : \mathbf{Type}$, and a family $\mathsf{El} : \mathsf{U} \to \mathbf{Type}$ that assigns, to each code corresponding to some type, the meta-theoretic type of its elements. The resulting definition is inductive-recursive because the inductive type of codes is defined simultaneously with the recursive function $\mathsf{El}$.

An example is the following definition of a small universe with bool and $\Pi$.

$$\begin{aligned} &\mathsf{data}\ \mathsf{U} : \mathbf{Type} && \mathsf{El} : \mathsf{U} \to \mathbf{Type} \\ &\quad \mathsf{bool} : \mathsf{U} && \mathsf{El}\ \mathsf{bool} :\equiv \mathbb{2} \\ &\quad \mathsf{pi} : (A : \mathsf{U}) \to (\mathsf{El}\ A \to \mathsf{U}) \to \mathsf{U} && \mathsf{El}\ (\mathsf{pi}\ A\ B) :\equiv (a : \mathsf{El}\ A) \to \mathsf{El}\ (B\ a) \end{aligned}$$

Induction-recursion is arguably a nice and natural way to define internal universes in type theory, however it is not always strictly required. We can translate basic instances of induction-recursion into inductive families using the equivalence of $I$-indexed families of types and types over $I$ (that is, $A : \mathbf{Type}$ with $A \to I$) [22].

In our case, we can encode $\mathsf{U}$ as an inductive type $\mathsf{inU}$ that *carves out* all types in $\mathbf{Type}$ that are in the image of $\mathsf{El}$. In other words, $\mathsf{inU}$ is a predicate that holds for any type that would have been obtained via $\mathsf{El}$ in the inductive-recursive definition. As $\mathsf{El}$ is indexed by the type of codes, the definition of $\mathsf{inU}$ quite expectedly reflects the inductive structure of codes.

$$\begin{aligned} &\mathsf{data}\ \mathsf{inU} : \mathbf{Type} \to \mathbf{Type}_1 \\ &\quad \mathsf{inBool} : \mathsf{in\text{-}U}\ \mathbb{2} \\ &\quad \mathsf{inPi} : \mathsf{inU}\ A \to ((a : A) \to \mathsf{inU}\ (B\ a)) \to \mathsf{inU}\ ((a : A) \to (B\ a)) \end{aligned}$$

$\mathsf{U}$ and $\mathsf{El}$ can be given by $\mathsf{U} :\equiv \Sigma\ (A : \mathbf{Type})\ (\mathsf{in\text{-}U}\ A)$ and $\mathsf{El} :\equiv \pi_1$.

Note that this construction gives rise to a universe in $\mathbf{Type}_1$, rather than $\mathbf{Type}$, since the definition of $\mathsf{U}$ quantifies over all possible types in $\mathbf{Type}$. Hence this kind of construction requires a metatheory with at least one universe.

#### 4.2 Inductive-recursive setoid universe

In this section we give a first definition of the setoid universe, as a direct generalization of the simple inductive-recursive definition just shown. We only consider a very small universe with bool type $\mathbb{2}$ and $\Pi$ for simplicity; a more realistic universe that includes more type formers can be found in the Agda formalization.

To construct the universe of setoids in the setoid model, we first of all need to define a type $\mathbb{U} : \mathrm{Ty}\ \Gamma$ for every $\Gamma : \mathrm{Con}$, and for every $A : \mathrm{Tm}\ \Gamma\ \mathbb{U}$ a type $\mathbb{E}l\ A : \mathrm{Ty}\ \Gamma$. Recalling Section 3, these are essentially record types made of several components. Since $\mathbb{U}$ is a closed type, it requires the same data of a setoid; in particular, we need a type of codes together with an equivalence relation reflecting equality of codes, in addition to proofs that these are indeed equivalence relations:

$$\begin{aligned} &\mathsf{data}\ \mathcal{U} : \mathbf{Type}_1 && \mathsf{refl}_{\mathcal{U}} : (A : \mathcal{U}) \to A \sim_{\mathcal{U}} A \\ &- \sim_{\mathcal{U}} - : \mathcal{U} \to \mathcal{U} \to \mathbf{Prop}_1 && \mathsf{sym}_{\mathcal{U}} : A \sim_{\mathcal{U}} B \to B \sim_{\mathcal{U}} A \\ & && \mathsf{trans}_{\mathcal{U}} : A \sim_{\mathcal{U}} B \to B \sim_{\mathcal{U}} C \to A \sim_{\mathcal{U}} C \end{aligned}$$

$\mathbb{E}l$ is given by a family of setoids indexed over the universe, that is, a way to assign to each code in the universe a carrier set and an equivalence relation.

$$\begin{aligned} \mathsf{El} &: \mathcal{U} \to \mathbf{Type} \\ - \vdash - \sim_{\mathsf{El}} - &: \{a\ a' : \mathcal{U}\} \to a \sim_{\mathcal{U}} a' \to \mathsf{El}\ a \to \mathsf{El}\ a' \to \mathbf{Prop} \end{aligned}$$

Note that $- \vdash - \sim_{\mathsf{El}} -$ is indexed over equality on the universe, because $\mathsf{El}$ is a displayed setoid over $\mathcal{U}$, hence in particular it must respect the setoid equality of $\mathcal{U}$. We also require data and proofs that make sure we get setoids out of $\mathsf{El}$:

$$\begin{aligned} \mathsf{refl}_{\mathsf{El}} &: (A : \mathcal{U})(x : \mathsf{El}\ A) \to \mathsf{refl}_{\mathcal{U}}\ A \vdash x \sim_{\mathsf{El}} x \\ \mathsf{sym}_{\mathsf{El}} &: p \vdash x \sim_{\mathsf{El}} x' \to \mathsf{sym}_{\mathcal{U}}\ p \vdash x' \sim_{\mathsf{El}} x \\ \mathsf{trans}_{\mathsf{El}} &: p \vdash x \sim_{\mathsf{El}} x' \to q \vdash x' \sim_{\mathsf{El}} x'' \to \mathsf{trans}_{\mathcal{U}}\ p\ q \vdash x \sim_{\mathsf{El}} x'' \\ \mathsf{coe}_{\mathsf{El}} &: A \sim_{\mathcal{U}} B \to \mathsf{El}\ A \to \mathsf{El}\ B \\ \mathsf{coh}_{\mathsf{El}} &: (p : A \sim_{\mathcal{U}} A')\ (x : \mathsf{El}\ A) \to p \vdash x \sim_{\mathsf{El}} \mathsf{coe}_{\mathsf{El}}\ p\ x \end{aligned}$$

We give an inductive definition of $\mathcal{U}$, mutually with a recursive definition of the 4 functions $- \sim_{\mathcal{U}} -$, $\mathsf{refl}_{\mathcal{U}}$, $\mathsf{El}$ and $- \vdash - \sim_{\mathsf{El}} -$. The other functions are then recursively defined: $\mathsf{refl}_{\mathsf{El}}$ alone, $\mathsf{sym}_{\mathcal{U}}$ and $\mathsf{sym}_{\mathsf{El}}$ mutually, $\mathsf{trans}_{\mathcal{U}}$, $\mathsf{trans}_{\mathsf{El}}$, $\mathsf{coe}_{\mathsf{El}}$ and $\mathsf{coh}_{\mathsf{El}}$ mutually. The whole construction is quite long, below we only show the more interesting definitions of $\mathcal{U}$ and $\mathsf{El}$:

$$\begin{aligned} &\mathsf{data}\ \mathcal{U} : \mathbf{Type}_1 \\ &\quad \mathsf{bool} : \mathcal{U} \\ &\quad \mathsf{pi} : (A : \mathcal{U})(B : \mathsf{El}\ A \to \mathcal{U}) \\ &\qquad \to (\{x\ x' : \mathsf{El}\ A\} \to \mathsf{refl}_{\mathcal{U}}\ A \vdash x \sim_{\mathsf{El}} x' \\ &\qquad\quad \to B\ x \sim_{\mathcal{U}} B\ x') \to \mathcal{U} \end{aligned}$$

$$\begin{aligned} &\mathsf{El}\ \mathsf{bool} :\equiv \mathbb{2} \\ &\mathsf{El}\ (\mathsf{pi}\ A\ B\ h) :\equiv \\ &\quad \Sigma\ (f : (a : \mathsf{El}\ A) \to \mathsf{El}\ (B\ a)) \\ &\qquad (\forall \{x\ x'\}(p : \mathsf{refl}_{\mathcal{U}}\ A \vdash x \sim_{\mathsf{El}} x') \\ &\qquad\quad \to h\ p \vdash f\ x \sim_{\mathsf{El}} f\ x') \end{aligned}$$

Note that in the definition of $\mathcal{U}$ we require that the family $B : \mathsf{El}\ A \to \mathcal{U}$ be a setoid morphism, respecting the setoid equalities involved. This choice is crucial for the definition of $\mathsf{El}$ to go through, in particular since we eliminate the code for $\Pi$ types into the setoid of functions that map equal elements to equal results. To state this mapping property we need to compare elements in different types, coming from applying $f$ to different arguments $x$ and $x'$. We know that $x$ and $x'$ are equal, but to conclude $B\ x \sim_{\mathcal{U}} B\ x'$ we need to know that $B$ respects setoid equality. This is exactly what we get from our definition of $\mathcal{U}$.

We can now give a full definition of the setoid universe, and of $\mathbb{E}l\ A$ for any $A : \mathrm{Tm}\ \Gamma\ \mathbb{U}$:

$$\begin{aligned} |\mathbb{U}| &:\equiv \lambda \gamma. \mathcal{U} & |\mathbb{E}l\ A| &:\equiv \lambda \gamma.\ \mathsf{El}\ (|A|\ \gamma) \\ \mathbb{U}^{\sim} &:\equiv \lambda p\, x\, y.\ x \sim_{\mathcal{U}} y & (\mathbb{E}l\ A)^{\sim} &:\equiv \lambda p\, x\, y.\ A^{\sim}\ p \vdash x \sim_{\mathsf{El}} y \\ \mathsf{refl}\ \mathbb{U} &:\equiv \mathsf{refl}_{\mathcal{U}} & \mathsf{refl}\ (\mathbb{E}l\ A) &:\equiv \mathsf{refl}_{\mathsf{El}} \\ &\ldots & &\ldots \\ \mathsf{coe}\ \mathbb{U} &:\equiv \lambda p\, a.\ a & \mathsf{coe}\ (\mathbb{E}l\ A) &:\equiv \lambda p.\ \mathsf{coe}_{\mathsf{El}}\ (A^{\sim}\ p) \\ \mathsf{coh}\ \mathbb{U} &:\equiv \lambda p.\ \mathsf{refl}_{\mathcal{U}} & \mathsf{coh}\ (\mathbb{E}l\ A) &:\equiv \lambda p.\ \mathsf{coh}_{\mathsf{El}}\ (A^{\sim}\ p) \end{aligned}$$

We can show that $\mathbb{U}$ is closed under $\Pi$ types and booleans, and satisfies $\mathbb{E}l\,(\mathsf{pi}\,A\,B) \equiv \Pi\,(\mathbb{E}l\,A)\,(\mathbb{E}l\,B)$ and $\mathbb{E}l\,\mathsf{bool} = \mathsf{Bool}$. The universe can be closed under more constructions if more codes are added to $\mathcal{U}$. This gives a complete definition of a universe of setoids, which is, however, inductive-recursive. Moreover, the kind of recursion involved in this definition is particularly complex, and not obviously reducible to well-understood notions of induction-recursion like the one described in [16]. In any case, we would like to avoid extending the metatheory with any form of induction-recursion in order to keep the metatheory as small and essential as possible.

In the next section we transform our current inductive-recursive definition to one that does not use induction-recursion. The way this is done is inspired by the well-known trick to eliminate induction-recursion described in Section 4.1, but modified in a novel way to account for the presence of **Prop**-valued types. To our knowledge, this is the first time this reduction method is applied to an inductive-recursive type of this kind.

#### 4.3 Inductive-inductive setoid universe

We will follow the method outlined in Section 4.1. In addition to $\mathsf{inU}$ for defining $\mathsf{U}$, we also introduce a family $\mathsf{inU}{\sim}$ of binary relations between types in the universe, from which we then define $- \sim_{\mathcal{U}} -$.

$$
\begin{aligned}
&\mathsf{data}\ \mathsf{inU} : \mathbf{Type} \to \mathbf{Type}_1\\
&\quad \mathsf{bool} : \mathsf{inU}\ \mathbb{2}\\
&\quad \pi : \mathsf{inU}{\sim}\ a\ a\ A_{\sim} \to (\forall\{x_0\ x_1\}(x_{01} : A_{\sim}\ x_0\ x_1) \to \mathsf{inU}{\sim}\ (b\ x_0)\ (b\ x_1)\ (B_{\sim}\ x_{01}))\\
&\qquad \to \mathsf{inU}\ (\Sigma\ (f : (x : A) \to B\ x)\\
&\qquad\qquad\qquad ((x_0\ x_1 : A)(x_{01} : A_{\sim}\ x_0\ x_1) \to B_{\sim}\ x_{01}\ (f\ x_0)\ (f\ x_1)))
\end{aligned}
$$

$$
\begin{aligned}
&\mathsf{data}\ \mathsf{inU}{\sim} : \{A\ A' : \mathbf{Type}\} \to \mathsf{inU}\ A \to \mathsf{inU}\ A' \to (A \to A' \to \mathbf{Prop}) \to \mathbf{Type}_1\\
&\quad \mathsf{bool}_{\sim} : \mathsf{inU}{\sim}\ \mathsf{bool}\ \mathsf{bool}\ (\lambda x_0\ x_1\ .\ x_0 \stackrel{?}{=}_{\mathbb{2}} x_1)\\
&\quad \pi_{\sim} :\ \ \{b_0 : (x_0 : A_0) \to \mathsf{inU}\ (B_0\ x_0)\}\{b_1 : (x_1 : A_1) \to \mathsf{inU}\ (B_1\ x_1)\}\\
&\qquad\quad \{a_{0\sim} : \mathsf{inU}{\sim}\ a_0\ a_0\ A_{0\sim}\}\{a_{1\sim} : \mathsf{inU}{\sim}\ a_1\ a_1\ A_{1\sim}\}\\
&\qquad\quad \{b_{0\sim} : \forall\{x_0\ x_1\}(x_{01} : A_{0\sim}\ x_0\ x_1) \to \mathsf{inU}{\sim}\ (b_0\ x_0)\ (b_0\ x_1)\ (B_{0\sim}\ x_{01})\}\\
&\qquad\quad \{b_{1\sim} : \forall\{x_0\ x_1\}(x_{01} : A_{1\sim}\ x_0\ x_1) \to \mathsf{inU}{\sim}\ (b_1\ x_0)\ (b_1\ x_1)\ (B_{1\sim}\ x_{01})\}\\
&\qquad \to \mathsf{inU}{\sim}\ a_0\ a_1\ A_{01\sim}\\
&\qquad \to (\forall\{x_0\ x_1\}(x_{01} : A_{01\sim}\ x_0\ x_1) \to \mathsf{inU}{\sim}\ (b_0\ x_0)\ (b_1\ x_1)\ (B_{01\sim}\ x_{01}))\\
&\qquad \to \mathsf{inU}{\sim}\ (\pi\ a_0\ a_{0\sim}\ b_0\ b_{0\sim})\ (\pi\ a_1\ a_{1\sim}\ b_1\ b_{1\sim})\\
&\qquad\qquad\qquad (\lambda f_0\ f_1\ .\ \forall(x_0\ x_1) \to A_{01\sim}\ x_0\ x_1 \to B_{01\sim}\ x_{01}\ (\pi_1\ f_0\ x_0)\ (\pi_1\ f_1\ x_1))
\end{aligned}
$$

Just as the role of $\mathsf{inU}$ is, as before, to classify all types that are image of $\mathsf{El}$, in the same way $\mathsf{inU}{\sim}\ a\ a'$ classifies all relations of type $A \to A' \to \mathbf{Prop}$ that are image of $- \vdash - \sim_{\mathsf{El}} -$, given proofs $a : \mathsf{inU}\ A, a' : \mathsf{inU}\ A'$. In particular, this definition of $\mathsf{inU}{\sim}$ states that the appropriate equivalence for boolean elements is the obvious syntactic equality $- \stackrel{?}{=}_{\mathbb{2}} -$, whereas functions are to be compared pointwise. Note that $\mathsf{inU}$ appears in the sort of $\mathsf{inU}{\sim}$. Since these types are mutually defined, they form an instance of *induction-induction*, a schema that allows the definition of a type mutually with other types that contain the first one in their signature [25].[9]

As in the universe example in Section 4.1, we now define $\mathcal{U}$ as a $\Sigma$ type, and $\mathsf{El}$ as the corresponding first projection.

$$\begin{aligned} &\mathcal{U} : \mathbf{Type}_1 && \mathsf{El} : \mathcal{U} \to \mathbf{Type} \\ &\mathcal{U} :\equiv \Sigma\ (X : \mathbf{Type})\ (\mathsf{inU}\ X) && \mathsf{El} :\equiv \pi_1 \end{aligned}$$

What is left now is to define the setoid equality relation on the universe, as well as the setoid equality relation on $\mathsf{El}\ A$ for any $A$ in $\mathcal{U}$. Two codes $A, B$ in the universe $\mathcal{U}$ are equal when there exists a setoid equivalence relation on their respective sets $\mathsf{El}\ A$ and $\mathsf{El}\ B$. Intuitively, since elements of a setoid are only ever compared to elements of the same setoid, this should only be possible if $A$ and $B$ are codes for the same setoid, that is, if $A \sim_{\mathcal{U}} B$. Existence and well-formedness of such relations is expressed via the type $\mathsf{inU}{\sim}$ just defined, hence we would expect $A \sim_{\mathcal{U}} B$ to be defined as follows:

$$(A, a) \sim_{\mathcal{U}} (B, b) :\equiv \Sigma\ (R : A \to B \to \mathbf{Prop})\ (\mathsf{inU}{\sim}\ a\ b\ R)$$

Unfortunately this definition only manages to capture the idea, but does not actually typecheck. In fact, $- \sim_{\mathcal{U}} -$ should be a $\mathbf{Prop}_1$-valued relation, so $A \sim_{\mathcal{U}} B$ should be a proposition. However, the $\Sigma$ type shown above clearly is not, since it quantifies over a type of relations, which is not a proposition. One possible solution is actually quite simple, and it just involves truncating the $\Sigma$ type above to force it to be in $\mathbf{Prop}_1$.

$$\begin{aligned} &- \sim_{\mathcal{U}} - : \mathcal{U} \to \mathcal{U} \to \mathbf{Prop}_1 \\ &(A, a) \sim_{\mathcal{U}} (B, b) :\equiv \|\Sigma\ (R : A \to B \to \mathbf{Prop})\ (\mathsf{inU}{\sim}\ a\ b\ R)\| \end{aligned}$$

We are now left to define the indexed equivalence relation on $\mathsf{El}$:

$$\begin{aligned} &- \vdash - \sim_{\mathsf{El}} - : \{A\ B : \mathsf{U}\} \to A\ \ \sim_{\mathcal{U}}\ \ B \to \mathsf{El}\ A \to \mathsf{El}\ B \to \mathbf{Prop} \\ &p \vdash x \sim_{\mathsf{El}} y :\equiv\ ? \end{aligned}$$

In the definition above, $p$ has type $\|\Sigma\ (R : \mathsf{El}\,A \to \mathsf{El}\,B \to \mathbf{Prop})\ (\ldots)\|$. If the type was not propositionally truncated, we could define $p \vdash x \sim_{\mathsf{El}} y$ by extracting the relation out of the first component of $p$, and apply it to $x, y$. That is, $p \vdash x \sim_{\mathsf{El}} y :\equiv \pi_1\, p\, x\, y$. This would make the definition of $- \sim_{\mathcal{U}} -$ and $- \vdash - \sim_{\mathsf{El}} -$ in line with how we defined $\mathcal{U}$ and $\mathsf{El}$.

However, this does not work in our case, since the type of $p$ *is* propositionally truncated, hence it cannot be eliminated to construct a proof-relevant object. Fortunately, we can work around this limitation by defining $p \vdash x \sim_{\mathsf{El}} y$ by induction on the codes $A\ B : \mathcal{U}$, in a way that ends up being logically equivalent to the proposition we would have obtained by $\pi_1\ p\ x\ y$ if there were no truncation.

[9] The main example of induction-induction is the intrinsic definition of a dependent type theory in type theory [6].

More precisely, we need to construct proofs that for any concrete $R$ and $\mathsf{inR}$, the types $|(R, \mathsf{inR})| \vdash x \sim_{\mathsf{El}} y$ and $R\ x\ y$ are logically equivalent. These in turn need to be defined mutually with $- \vdash - \sim_{\mathsf{El}} -$. We direct the interested reader to the Agda formalization for the full details of these definitions, as they are quite involved.

The full definition of the universe is concluded with the remaining definitions, like $\mathsf{refl}_{\mathcal{U}}, \mathsf{refl}_{\mathsf{El}}$, etc., which can be adapted from their IR counterparts more or less straightforwardly. The final result does not use induction-recursion, but it is nevertheless an instance of infinitary induction-induction. The ability to define arbitrary, infinitary inductive-inductive types clashes, again, with our objective of keeping the metatheory as small and simple as possible. The next step is therefore to reduce this inductive-inductive universe to one that does not require (infinitary) induction-induction.

#### 4.4 Inductive setoid universe

This section encodes the inductive-inductive universe of setoids from the previous section without assuming arbitrary inductive-inductive definitions in the metatheory.

Before turning our attention to the setoid universe, we recall the known, systematic method to reduce finitary inductive-inductive types to inductive families.

**Reducing finitary induction-induction** It is known that finitary inductive-inductive definitions can be reduced to inductive families [8,7,21]. To illustrate the idea, let us consider a well-known example of a finitary inductive-inductive type, the intrinsic encoding of type theory in type theory itself. Actually, we only consider the type of contexts $\mathsf{Con} : \mathbf{Type}$ and the type of types $\mathsf{Ty} : \mathsf{Con} \to \mathbf{Type}$; since the latter is indexed over the former, this is already an example of induction-induction.

Contexts in $\mathsf{Con}$ are formed out of empty contexts $\bullet$ and context extension $-,-$. Types in $\mathsf{Ty}$ are either the base type $\iota$ or $\Pi$ types.

$$\bullet : \mathsf{Con} \qquad\qquad \iota : (\Gamma : \mathsf{Con}) \to \mathsf{Ty}\ \Gamma$$
$$-,- : (\Gamma : \mathsf{Con}) \to \mathsf{Ty}\ \Gamma \to \mathsf{Con} \qquad\qquad \Pi : \{\Gamma : \mathsf{Con}\}(A : \mathsf{Ty}\ \Gamma) \to \mathsf{Ty}\ (\Gamma, A) \to \mathsf{Ty}\ \Gamma$$

The general method to eliminate induction-induction is to split the original inductive-inductive types into a type of codes and associated well-formedness predicates. In our $\mathsf{Con}/\mathsf{Ty}$ example, these would be respectively given by codes $\mathsf{Con}_0, \mathsf{Ty}_0 : \mathbf{Type}$ and predicates $\mathsf{Con}_1 : \mathsf{Con}_0 \to \mathbf{Type}, \mathsf{Ty}_1 : \mathsf{Con}_0 \to \mathsf{Ty}_0 \to \mathbf{Type}$.

The definition of the codes and predicate types follows that of the original inductive-inductive type, and can be derived systematically from it. More importantly, they can be defined without induction-induction, since although $\mathsf{Con}_0$ and $\mathsf{Ty}_0$ are defined mutually, their sorts are not indexed.

$$\begin{aligned}
\bullet_0 &: \mathsf{Con}_0 \\
-,_0 - &: \mathsf{Con}_0 \to \mathsf{Ty}_0 \to \mathsf{Con}_0 \\
\iota_0 &: \mathsf{Con}_0 \to \mathsf{Ty}_0 \\
\Pi_0 &: \mathsf{Con}_0 \to \mathsf{Ty}_0 \to \mathsf{Ty}_0 \to \mathsf{Ty}_0
\end{aligned}$$

$$\begin{aligned}
\bullet_1 &: \mathsf{Con}_1\ \bullet_0 \\
-,_1 - &: \forall\{\Gamma_0\ A_0\} \to \mathsf{Con}_1\ \Gamma_0 \to \mathsf{Ty}_1\ \Gamma_0\ A_0 \\
&\quad \to \mathsf{Con}_1\ (\Gamma_0 ,_0 A_0) \\
\iota_1 &: \forall\{\Gamma_0\} \to \mathsf{Con}_1\ \Gamma_0 \to \mathsf{Ty}_1\ \Gamma_0\ (\iota_0\ \Gamma_0) \\
\Pi_1 &: \forall\{\Gamma_0\ A_0\ B_0\} \to \mathsf{Con}_1\ \Gamma_0 \\
&\quad \to \mathsf{Ty}_1\ \Gamma_0\ A_0 \to \mathsf{Ty}_1\ (\Gamma_0 ,_0 A_0)\ B_0 \\
&\quad \to \mathsf{Ty}_1\ \Gamma_0\ (\Pi_0\ \Gamma_0\ A_0\ B_0)
\end{aligned}$$

We can recover the original inductive-inductive type as $\mathsf{Con} :\equiv \Sigma\ (\Gamma_0 : \mathsf{Con}_0)\ (\mathsf{Con}_1\ \Gamma_0)$ and $\mathsf{Ty}\ \Gamma :\equiv \Sigma\ (A_0 : \mathsf{Ty}_0)\ (\mathsf{Ty}_1\ (\pi_1\ \Gamma)\ A_0)$. Recovering the constructors is straightforward:

$$\begin{aligned}
&\bullet && :\equiv (\bullet_0, \bullet_1) \\
&(\Gamma_0, \Gamma_1), (A_0, A_1) && :\equiv ((\Gamma_0 ,_0 A_0), (\Gamma_1 ,_1 A_1)) \\
&\iota\ (\Gamma_0, \Gamma_1) && :\equiv (\iota_0\ \Gamma_0, \iota_1\ \Gamma_1) \\
&\Pi\ \{\Gamma_0, \Gamma_1\}(A_0, A_1)(B_0, B_1) && :\equiv (\Pi_0\ \Gamma_0\ A_0\ B_0, \Pi_1\ \Gamma_1\ A_1\ B_1)
\end{aligned}$$

Finally, we can define eliminators/induction principles for Con and Ty as just defined, by induction on the well-typing predicates.

Following [25], we distinguish two versions of the eliminator: the *simple* and the *general* one. Note that this is orthogonal to the distinction between non-dependent and dependent eliminators, from which we only consider the latter. The motives for the simple eliminator are $C' : \mathsf{Con} \to \mathbf{Type}$, $T' : (\Gamma : \mathsf{Con})(A : \mathsf{Ty}\,\Gamma) \to \mathbf{Type}$ and the eliminators themselves have the following signatures:

$$\mathsf{elim}'_{\mathsf{Con}} : (\Gamma : \mathsf{Con}) \to C'\ \Gamma \qquad \mathsf{elim}'_{\mathsf{Ty}} : \forall\{\Gamma\}(A : \mathsf{Ty}\ \Gamma) \to T'\ \Gamma\ A$$

In the case of the general eliminator, the motive for Ty depends on the motive for Con, making the two eliminators *recursive-recursive* functions. For motives $C : \mathsf{Con} \to \mathbf{Type}$ and $T : (\Gamma : \mathsf{Con}) \to \mathsf{Ty}\ \Gamma \to C\ \Gamma \to \mathbf{Type}$ the signatures are:

$$\mathsf{elim}_{\mathsf{Con}} : (\Gamma : \mathsf{Con}) \to C\ \Gamma \qquad \mathsf{elim}_{\mathsf{Ty}} : \forall\{\Gamma\}(A : \mathsf{Ty}\ \Gamma) \to T\ \Gamma\ A\ (\mathsf{elim}_{\mathsf{Con}}\ \Gamma)$$

The general eliminators can be derived from our encoding of Con and Ty via untyped codes and well-typing predicates. The way to do it is to first define the graph of the eliminators in the form of inductively-generated relations:

$$\begin{aligned}
&\mathsf{data\ R\text{-}Con} : (\Gamma : \mathsf{Con}) \to C\ \Gamma \to \mathbf{Type} \\
&\mathsf{data\ R\text{-}Ty}\ \ : \{\Gamma : \mathsf{Con}\}(A : \mathsf{Ty}\ \Gamma)(\gamma : C\ \Gamma) \to T\ \Gamma\ A\ \gamma \to \mathbf{Type}
\end{aligned}$$

The next step is to prove that these relations are functional, by induction on the untyped codes $\mathsf{Con}_0$ and $\mathsf{Ty}_0$ [21]. From this result, defining the eliminators is immediate.

**Reducing the setoid universe** The reduction described in the previous section works generically for an arbitrary finitary inductive-inductive type, thus giving a systematic way to reduce finitary inductive-inductive definitions to inductive families. However, it is not clear whether this method extends to *infinitary* induction-induction, of which the setoid universe defined in Section 4.3 is an instance. Of course, the absence of a general reduction method does not mean that we cannot reduce particular concrete instances of infinitary induction-induction, which is exactly what we hope for our universe construction.

The obvious challenge in successfully completing this reduction is to avoid the need for extensionality in the metatheory. In fact, consider the simple infinitary inductive-inductive type obtained from the previous Con/Ty example by replacing the finitary constructor $\Pi$ with an infinitary one: $\Pi : \{\Gamma : \mathsf{Con}\} \to (\mathbb{N} \to \mathsf{Ty}\ \Gamma) \to \mathsf{Ty}\ \Gamma$. Already with this simple example, we run into problems as soon as we try to define the eliminator. One issue is that the definition of the eliminator relies on a proof that the well-typing predicates $\mathsf{inU}_1, \mathsf{inU}\!\sim_1$ are propositional, that is, any two of their elements are equal. Without further assumptions this proof can only be done by induction, and requires function extensionality since these predicates include higher-order constructors.

One way to get around this is to define the well-typing predicates as **Prop**-valued families, rather than in **Type**:

$$
\begin{aligned}
&\mathsf{data}\ \mathsf{inU}_0 && : \mathbf{Type} \to \mathbf{Type}_1\\
&\mathsf{data}\ \mathsf{inU}\!\sim_0 && : \{A\ A' : \mathbf{Type}\} \to (A \to A' \to \mathbf{Prop}) \to \mathbf{Type}_1\\
&\mathsf{data}\ \mathsf{inU}_1 && : (A : \mathbf{Type}) \to \mathsf{inU}_0\ A \to \mathbf{Prop}_1\\
&\mathsf{data}\ \mathsf{inU}\!\sim_1 && : \{A\ A' : \mathbf{Type}\} \to (R : A \to A' \to \mathbf{Prop}) \to \mathsf{inU}\!\sim_0\ R \to \mathbf{Prop}_1
\end{aligned}
$$

Using **Prop** avoids the issue of proving propositionality altogether, since the predicates are now propositional by definition. However, it introduces a different issue: $\mathsf{inU}_1$ and $\mathsf{inU}\!\sim_1$ give rise to equational constraints on their indices, in the form of proofs of the **Prop**-valued identity type. The definition of the eliminators for $\mathsf{inU}$ and $\mathsf{inU}\!\sim$ relies on the ability to transport along these proofs, hence the need to extend our metatheory with a primitive, strong form of transport for Id.[10]

Having **Prop** and a strong transport principle does help to some extent. However, we would still need extensionality to derive the general eliminators for $\mathsf{inU}$ and $\mathsf{inU}\!\sim$. In fact, as explained in the previous section, to derive the general recursive-recursive eliminators we need to prove that the corresponding graph relations are functional, which cannot be done without funext.

Luckily, the *simple* elimination principle is sufficient for our purposes: all functions described in Section 4.3 can be defined just using the simple eliminator without recursion-recursion. The simple eliminator itself can be defined by pattern matching on the untyped codes, and does not require extensionality or any extra principles beyond strong transport.

Once the inductive encoding of the inductive-inductive universe is done, the setoid universe can be defined just as in Section 4.3.

[10] Note that this issue cannot be solved by expressing the equational constraints with an identity type in **Type**, since the well-typing predicates force it to necessarily be in **Prop**.

### 5 Conclusions and further work

We have described the construction of a universe of setoids in the setoid model of type theory; this is given in several steps, first as an inductive-recursive definition, then as an inductive-inductive definition, and finally as an inductive type. Every encoding is obtained from the previous by adapting known data type transformation methods in a novel way that accounts for the peculiarities of our construction. In [5] we present rules for SetTT, clearly these rules need to be extended by the rules for a universe reflecting the semantics presented here.

It is known that finitary IITs can be reduced to inductive types in an extensional setting [21]. In our paper we reduce an infinitary IIT to inductive types in an intensional setting. In the future, we would like to investigate whether this reduction can be generalised to arbitrary infinitary IITs.

In contrast to the inductive-recursive and inductive-inductive versions of the universe, the inductive definition relies on a metatheory with a strong transport rule. As future work, we would like to prove normalization for this metatheory since previous work in this respect [2] seems to suggest that is represents a non-trivial addition.

Another question regards the relationship between SeTT [5] and XTT [28]. Both systems are syntactic representations of the setoid model with similar design choices, like definitional proof-irrelevance. We would like to know whether their respective notions of models are equivalent, that is, if we can obtain an XTT model from a SeTT model, and vice versa. Since XTT universes support universe induction, for one direction we would need to extend our own universe with the same principle (see discussion in Section 3 and the previous paragraph). Thus a related question is whether our encodings of the setoid universe can support universe induction. A further question is whether this mapping of models is functorial.

Groupoids can be regarded as generalized setoids. In the future we would like to design a type theory internalizing the groupoid model of type theory [20], in the same way that SeTT represents a syntax for the setoid model. A further question is whether such "groupoid type theory" can be justified, similarly to SeTT, via a syntactic translation, perhaps with SeTT itself as the target theory.
